# Comparing AI-Driven and Heart Team Decision-Making in Multivessel Coronary Artery Disease

**DOI:** 10.3390/jcm14134452

**Published:** 2025-06-23

**Authors:** Stefano Migliaro, Roberto Celotto, Romina Teliti, Simona Mariani, Luca Altamura, Fabrizio Tomai

**Affiliations:** Department of Cardiovascular Sciences, European Hospital and Aurelia Hospital, 00165 Rome, Italy; migliaro.stefano@gmail.com (S.M.); celott.roberto@yahoo.it (R.C.); telitiromina@yahoo.it (R.T.); simona.mariani.md@gmail.com (S.M.); lucaaltamura@gmail.com (L.A.)

**Keywords:** multivessel coronary artery disease (CAD), heart team (HT), coronary artery bypass grafting (CABG), percutaneous coronary intervention (PCI), artificial intelligence (AI)

## Abstract

**Background/Objectives**: Multivessel coronary artery disease (CAD) remains a challenging condition requiring multidisciplinary decision-making, particularly when determining between percutaneous coronary intervention (PCI) and coronary artery bypass grafting (CABG). Recent advancements in artificial intelligence (AI), particularly generative language models like ChatGPT, present an opportunity to assist in the decision-making process. However, their ability to replicate human clinical judgment in complex scenarios, such as multivessel CAD, remains untested. **Methods**: The aim of this study was to evaluate the concordance between recommendations from AI (ChatGPT) and those from heart team (HT) in the management of multivessel CAD, with a focus on comparing treatment strategies such as PCI and CABG. A retrospective observational study was conducted on 137 patients with multivessel CAD, discussed at multidisciplinary HT meetings in 2024. Standardized clinical vignettes, including clinical and anatomical data, were presented to ChatGPT for treatment recommendations. The AI’s responses were compared with the HT’s decisions regarding PCI or CABG. Statistical analysis was performed to assess the level of agreement and predictive value of ChatGPT’s recommendations. **Results**: ChatGPT achieved an overall accuracy of 65% in its recommendations. The agreement rate was higher for CABG (82.4%) than for PCI (44.4%). Discordance was identified in 48 patients, with a notable bias towards recommending CABG. Factors such as age, diabetes, and chronic kidney disease were predictors of discordance, although no significant factors emerged for the PCI or CABG subgroups. **Conclusions**: AI, particularly ChatGPT, demonstrated modest concordance with HT decisions in the management of multivessel CAD, especially favoring CABG. While AI offers potential as a decision-support tool, its current limitations highlight the continued need for human clinical judgment in complex cases. Further research is required to optimize AI integration into clinical decision-making frameworks.

## 1. Introduction

Contemporary cardiology practice has increasingly embraced a multidisciplinary approach, with the heart team (HT) serving as a central reference point for addressing a wide range of indications and conditions. With the improvement in percutaneous treatment techniques, clinical guidelines have upgraded the role of HT in the decision-making process of many conditions. The initial evidence supporting the clinical utility of HT discussions first emerged in the context of multivessel coronary artery disease (CAD). This area has traditionally represented a “grey zone”, where both surgical and percutaneous revascularization approaches may be viable and offer distinct advantages and limitations [1].

A thorough evaluation of factors such as clinical presentation, comorbidities, coronary anatomy, social conditions, and patient preference is, therefore, critical, requiring the HT to serve as the cornerstone of the entire therapeutic pathway within the modern cardiology ward [2].

Despite their crucial role, HT meetings can be challenging to organize and may suffer from partial attendance. Furthermore, in high-volume centers, the sheer number of cases requiring discussion often leads to expedited analyses. This time pressure can result in superficial evaluations that fail to explore every nuance of a clinical case or, conversely, focus on less critical aspects of the scenario. Such limitations can ultimately compromise the effectiveness and utility of HT meetings.

In this context, artificial intelligence (AI), particularly generative large language models like ChatGPT 4.0, has emerged as a potentially game-changing tool [3]. These models excel at processing and synthesizing vast datasets, potentially enabling them to deliver rapid, evidence-based recommendations with high precision. In particular, the transformer architecture, introduced by Google in 2017, allows the computer to process words and their relationships in parallel, in order to create the most coherent answer possible [4].

Few studies have investigated the ability of AI to deliver valuable recommendations in complex clinical scenarios. Notably, prior research has examined AI’s role in the management of valvular heart diseases, particularly severe aortic stenosis, showing encouraging data with a good concordance with HT decisions [5]. However, multivessel CAD presents unique challenges due to its intricate interplay of anatomical, procedural, and clinical considerations, making it a more demanding context for AI-assisted decision-making.

This study examines the concordance between ChatGPT’s recommendations and those of HTs in the management of multivessel CAD. By analyzing discrepancies, the study seeks to evaluate ChatGPT’s capacity to replicate nuanced clinical decision-making and to explore its potential as a complementary tool for decision support.

## 2. Materials and Methods

### 2.1. Study Population

The data were retrospectively collected from clinical records to include patients discussed at a multidisciplinary meeting in a high-volume Italian center in 2024. Our study was conducted in accordance with the principles of the Declaration of Helsinki. The study included all the patients with stable or stabilized multivessel CAD undergoing multidisciplinary HT discussion. Those patients receiving indication to medical management (without coronary revascularization) were excluded from the study.

### 2.2. Clinical Evaluation and Heart Team Meetings

HT meetings were conducted regularly and included interventional cardiologists, imaging specialists, cardiac surgeons, a vascular surgeon, an anesthesiologist, and a geriatrician. Each case underwent a comprehensive evaluation of clinical, echocardiographic, and anatomical parameters. This collaborative decision-making process consistently ensured the selection of the most appropriate management strategy for each patient. As this is a retrospective study, HT decisions were intrinsically blinded to any AI-derived recommendation.

### 2.3. Clinical Vignette Presented to ChatGPT

**Figure 1:**
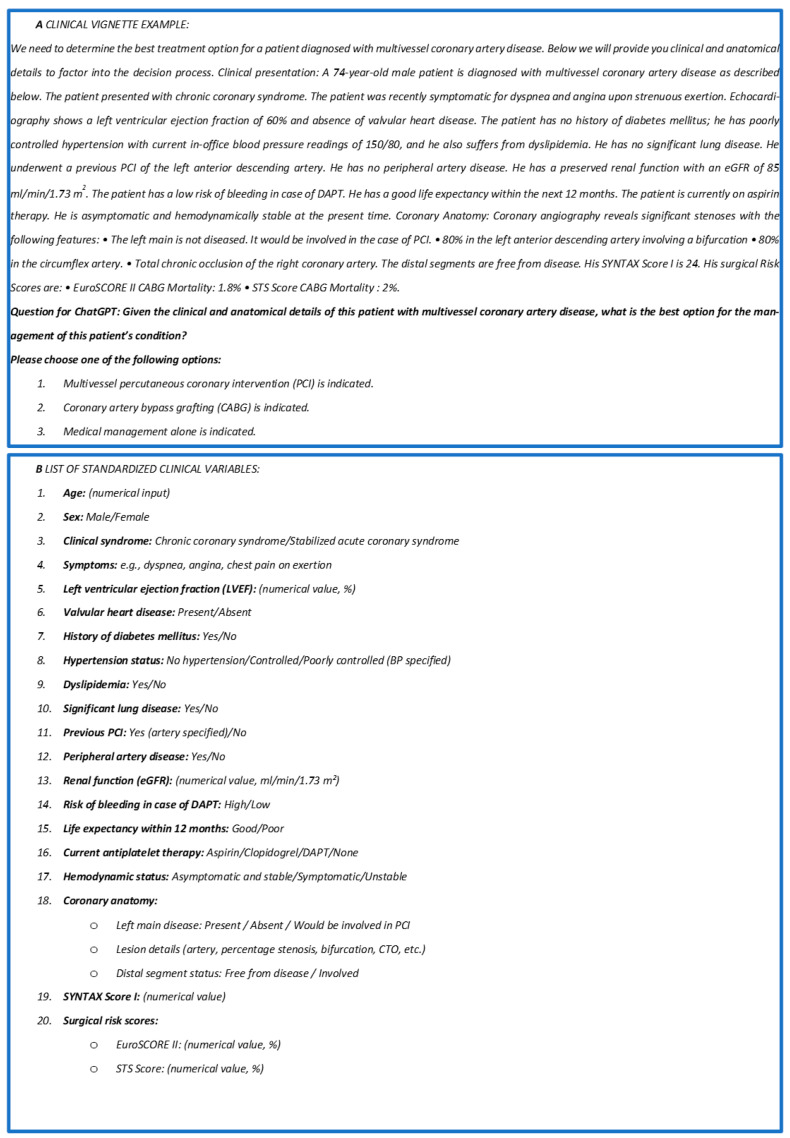
(**A**) Example of standardized text that was used for each patient and submitted to ChatGPT; (**B**) list of the standardized clinical variables submitted to the AI. PCI: percutaneous coronary intervention; eGFR: estimated glomerular filtration rate; DAPT: dual antiplatelet therapy; LVEF: left ventricular ejection fraction; SYNTAX Score: Synergy Between PCI With Taxus and Cardiac Surgery Score; EuroSCORE II: European System for Cardiac Operative Risk Evaluation II; STS Score: Society of Thoracic Surgeons Score.

For each patient, a standardized clinical vignette was created, incorporating key clinical variables alongside a detailed description of the coronary anatomy and major anatomical (SYNTAX) and procedural risk scores (EuroSCORE II, STS Score). The vignette structure, phrasing, and sequence were identical for all cases, with only objective clinical and anatomical variables modified according to the individual patient. All non-numerical variables were selected from a predetermined set of clinically relevant options, rather than being freely modified, to minimize subjectivity and ensure consistency. This approach ensured that each vignette reflected the clinical factors routinely considered by heart teams when choosing between PCI and CABG, thus aligning the AI prompt with real-world multidisciplinary practice. Critical data, including the presence of bifurcations, left main disease or involvement by stenting, calcifications, and chronic total occlusions, were highlighted and meticulously documented. Treatment options were analyzed and processed using the artificial intelligence-powered chatbot known as the Chat Generative Pre-trained Transformer (ChatGPT), version 4.0 Omnia (GPT-4o), developed by OpenAI (OpenAI, L.L.C., San Francisco, CA). Social conditions were classified as poor if two or more of the following criteria were met: education below the second level (middle school), lack of fluency in Italian, absence of stable housing, poor hygienic conditions, unstable employment, or lack of living relatives. Regarding the evaluation of carotid arteries, stenoses < 50% were classified as non-significant. For each patient, two possible treatment strategies—percutaneous coronary intervention (PCI) or coronary artery bypass grafting (CABG)—were proposed to the AI for consideration. An example of a clinical vignette presented to ChatGPT, followed by the list of included variables and possible variable “status”, is shown in Figure 1A,B.

### 2.4. Outcomes

The interrogation of ChatGPT was performed by a physician blinded to the HT decision; the first response provided by the AI was accepted as valid without further interaction or discussion. Each case was presented to ChatGPT in a dedicated window to prevent the inheritance of data from previous cases. The AI’s responses were subsequently compared to the treatment recommendations provided by the HT. The primary outcome was the level of agreement between ChatGPT and the HT regarding the optimal treatment strategy (PCI or CABG).

### 2.5. Statistical Analysis

The data were summarized using descriptive statistics, with mean ± standard deviation (SD) for normally distributed continuous variables and median (interquartile range [IQR]) for non-normally distributed continuous variables. Frequencies with percentages were used for categorical variables. Group comparison was performed using Student’s *t*-test, Chi^2^ analysis, and multivariable logistic regression with and without bootstrapping. Sensitivity, specificity, positive and negative predictive values, and accuracy were calculated. All the analyses were carried out using SPSS Statistics version 29 (IBM).

## 3. Results

### 3.1. Patient Characteristics

**Figure 2:**
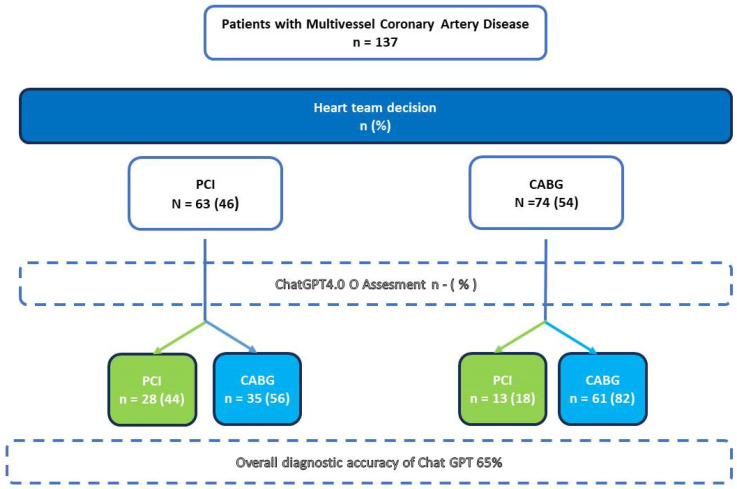
Reclassification of treatment decisions for patients with multivessel coronary artery disease based on both heart team (HT) and ChatGPT assessments; PCI: percutaneous coronary intervention; CABG: coronary artery bypass grafting; ChatGPT: Chat Generative Pre-trained Transformer; HT: heart team.

A total of 137 consecutive patients with multivessel CAD suitable for coronary revascularization discussed during HT meetings were included in the study. Table 1 reports the main characteristics of the overall cohort. The mean age was 70.3 ± 8.6 years, with most patients presenting with chronic coronary syndromes (n =115, 83.9%) and preserved left ventricular ejection fraction (LVEF) (mean: 54.3% ± 8.2). A significant proportion of the cohort had diabetes mellitus (n = 58, 42.3%). Evidence of ischemia on non-invasive evaluation was available in nearly half of the population (n = 65, 47.4%). Only a minority of patients had previously undergone PCI (n = 22, 16.1%) or had significant carotid disease (n = 21, 15.3%). Coronary angiography revealed widespread significant CAD. The surgical risk, assessed using the STS Score mortality and EuroSCORE II, averaged 2.1 ± 2.2% and 2.1 ± 2.1%, respectively. Left main disease was identified in one in five patients (n = 28, 20.4%), while two-vessel disease was rare (n = 7, 5.1%). A considerable proportion of patients had at least one chronically occluded coronary vessel (n = 55, 40.2%). The mean SYNTAX score in the overall cohort was 25.2 ± 7.7. Main treatment recommendations provided by the HT and by the AI are summarized in Figure 2.

### 3.2. Heart Team Decisions

According to the HT assessment, 63 (46%) patients were deemed eligible for PCI, while CABG was considered the preferable treatment option in 74 (54%) cases. Table 1 provides a detailed summary of the baseline characteristics of the overall study population, as well as those of each subgroup.

### 3.3. ChatGPT Decisions

The AI model provided a valid recommendation for all 137 patients in the cohort. For the majority of patients (n = 97, 70.1%), ChatGPT recommended CABG. The model achieved an overall accuracy of 65% and demonstrated a moderate Kappa coefficient of 0.276. The agreement rate (sensitivity) was relatively low for patients eligible for PCI (44.4%) but was higher for those deemed suitable for CABG (82.4%). The chatbot did not perform well in terms of positive predictive value (68.3%) or negative predictive value (63.5%) (Table 2).

GPT-4o “misclassified” a total of 48 (35%) patients, recommending CABG instead of PCI for 35 patients and PCI instead of CABG for 13 patients. On univariate analysis, there were only a minority of factors that, taken alone, may be responsible for recommendation disagreement: when comparing the HT-indicated PCI cohort with the GPT-indicated PCI (theoretical) cohort (Table 3), the only barely significant differences were a slightly lower ejection fraction in the HT group (51 ± 10% vs. 56 ± 7%, *p* = 0.020) and a higher prevalence of hypertension (95.2% vs. 82.9%, *p* = 0.047). This was similar to the HT-indicated CABG cohort versus the ChatGPT-indicated CABG cohort (Table 4), and LVEF was slightly higher in the former (56.5 ± 8.45% vs. 53.5 ± 10.4%, *p* = 0.043). These differences are likely not clinically significant.

After selecting some of the most relevant clinical variables—based on physician judgment—as inputs for the multivariate logistic regression, the predictors of overall discordance between AI and HT decisions identified were age, diabetes, and chronic kidney disease (Table 5).

To confirm the robustness of these findings, a bootstrapped logistic regression analysis with 5000 iterations was performed (Table 6). This analysis corroborated age and reduced renal function (eGFR ≤ 35 mL/min/1.73 m^2^) as independent predictors of discordance between AI and HT decisions (OR = 0.072, *p* = 0.034; OR = −1.660, *p* = 0.019, respectively). Additionally, a higher STS Score for mortality was significantly associated with reduced likelihood of discordance (OR = −0.564, *p* = 0.020), suggesting that in more complex or high-risk patients, AI recommendations tend to align more closely with heart team choices.

## 4. Discussion

This paper explores the potential application of AI to aid clinical decision-making in the management of multivessel CAD. Our main findings are as follows:

1. ChatGPT demonstrated only modest agreement with HT decision-making.
2. No major identifiable clinical factors were found to drive treatment assignment by the AI model.
3. ChatGPT’s recommendations were predominantly skewed towards CABG for most of the cohort.

The past couple of years have witnessed the explosive rise of general-purpose AI systems. Unlike earlier iterations, these technologies are not confined to selected, pre-specified tasks; instead, they are capable of addressing a vast array of problems across virtually every domain of human activity. Since late 2022, there has been a marked surge in interest and activity surrounding AI, with major corporations racing to integrate its capabilities into their services [6].

In recent years, practical applications of AI have already demonstrated substantial value in the field of cardiology. For example, AI algorithms have achieved high accuracy in the detection of atrial fibrillation from ECGs and wearable devices, supported advanced mapping and outcome prediction in catheter ablation for atrial fibrillation, and improved the identification of heart failure patients most likely to benefit from cardiac resynchronization therapy through the integration of clinical and imaging data. These examples highlight the growing utility of AI as an adjunct in cardiology, particularly in domains where large volumes of complex data can be synthesized to guide more precise and personalized clinical decisions [7].

In the field of cardiovascular interventions, the potential applications of AI have not been extensively explored. To our knowledge, two recently published studies have previously investigated the role of AI in clinical decision-making. Specifically, one study evaluated the ability of AI to classify treatment indications for severe aortic stenosis [5], showing that ChatGPT’s decisions were consistent with those of the HT in a large proportion of cases. Another piece of research, more similar to our study, performed a comparison of the performance of the AI versus the HT in the setting of coronary revascularization, showing also a decent agreement [8].

In our analysis, the performance of the AI was suboptimal, with an overall accuracy of 65%, which was notably worse than the results observed in the study on aortic stenosis [5]. This was particularly evident for patients receiving indication to PCI, for whom the AI achieved concordance in less than half of the cases (44.4%), compared to significantly better results for patients receiving indication to CABG (82.4%). Indeed, while decision-making in valvular disease typically relies on more abstractable and structured data, allowing for easier alignment with straightforward guideline applications, the same does not hold true for CAD. In fact, in the case of aortic stenosis, transcatheter aortic valve implantation is widely recognized as a first-line therapy in most cases, simplifying the decision-making process and reducing the variability in treatment recommendations.

Interestingly, our study reports a notable discrepancy in overall accuracy compared to the 82% rate described in the other published work in the same setting [8]; moreover, unlike our observations, that study demonstrated improved AI performance within specific patient subgroups. Several factors may account for these differences: variations in prompt formulation, despite providing identical information to the AI, could influence the output; differences in the clinical characteristics of the patient populations may also affect model performance; the other study employed version 4 of the model, whereas we utilized the newer version 4-o; and finally, discrepancies in the decision attitude of the HT, shaped by institutional experience and resource availability, might have influenced the preference for PCI or CABG as the optimal treatment strategy. Regardless of the underlying causes of this disagreement, it is essential to emphasize that even an 82% accuracy rate falls short of the standards typically expected for medical devices. In our opinion, these findings reflect a fundamental limitation of the AI’s response-generation mechanism.

AI models generate responses by evaluating the statistical relationships between words and their contexts, producing outputs based on the most likely word sequences within their training database. Given that the majority of the scientific literature on multivessel CAD has historically favored CABG over PCI, it was anticipated that the AI would exhibit a strong inclination toward recommending surgical revascularization. That said, in our study, it remains unclear which specific factors were most influential in skewing the AI model’s decisions toward CABG. The standard logistic regression model identified age, diabetes, and chronic kidney disease (CKD) as predictors of disagreement, while the bootstrapped analysis confirmed age and CKD as significant determinants of discordance but identified also the clinical risk scores (namely STS and EuroSCORE), that narrowly missed the threshold for significancy at the initial analysis. Notably, the SYNTAX score did not achieve statistical significance at the standard or at the bootstrapped logistic regression. These findings further highlight the differences in data analysis and interpretation by the HT compared to the AI; while the group of experts leveraged their indication on a multifaceted patient-tailored analysis, the AI strongly grounded its recommendation on the numerical risk scores, which can be more easily algorithmically processed.

On the other hand, it is precisely in this context that human evaluation demonstrates its superiority. Experienced clinicians bring a nuanced understanding that extends beyond the isolated results of primary endpoints of clinical trials, factoring both measurable and non-measurable variables into the decision-making process. Furthermore, clinicians consider a variety of contextual factors, including their own expertise with certain anatomies, the availability of specific devices, and center-specific logistical constraints that may significantly impact the outcomes of both CABG and PCI.

In our opinion, the primary value of the HT lies in its ability to ensure comparable outcomes while prioritizing the least invasive and most patient-centered treatment. This synthesis of technical and scientific knowledge with human experience ensures a holistic approach that AI systems currently lack. On the other hand, one potential advantage of integrating AI-based decision support into clinical workflows is the reduction in time required for case evaluation. In the conventional HT process, approximately 10 min is needed for an individual clinician to retrieve and organize case data, followed by around 5 min for presentation and 10 min for discussion and consensus-building, totaling about 25 min per case. In contrast, the AI approach requires a similar initial data collection phase (about 10 min), but the subsequent submission to the AI and review of its recommendation are almost instantaneous. While these estimates are theoretical and depend on the case complexity and institutional workflow, they suggest that AI could streamline decision-making and reduce overall time investment per case. Prospective studies will be necessary to confirm and quantify these potential efficiency gains.

A critical limitation of conversation-based large language models available at the time of our study is the inability to directly interpret structured imaging data or capture the subtle anatomical details and technical considerations that emerge from direct image review. Consequently, aspects such as lesion complexity, vessel characteristics, and the feasibility of different interventional approaches, which are integral to HT decision-making, may not be fully represented or weighted in the AI’s assessment. Since ChatGPT-4o was the only AI model tested in this study, we cannot make any statement on the performance of other models.

Another limitation of AI in clinical decision-making, as recently shown, is the dependency on subtle changes in prompt phrasing, variations in the assumed role of the user (e.g., user vs. physician), and differences between free and paid versions or models of the software. All these factors can produce substantial variations in the AI output [9].

Table 7 offers a comprehensive comparison of the strengths and limitations of HT and AI.

Beyond the technical limitations previously discussed, significant ethical, legal, and operational challenges persist in the integration of AI-driven decision support into the clinical management of multivessel CAD. From an ethical perspective, artificial intelligence cannot incorporate empathy, nuanced understanding of personal context, or the lived intentions and values of individual patients—dimensions that often prove decisive in complex clinical decisions. There is a risk that increased reliance on AI could depersonalize care and shift responsibility away from the clinical team, potentially undermining the physician’s central role as patient advocate and increasing the risk of “moral distancing” from critical choices.

Medico-legal considerations are equally important. The implementation of fully or partially AI-guided recommendations would require dedicated legal frameworks, as current systems are not designed to address liability for adverse outcomes that result from algorithm-generated advice, particularly in cases of disagreement between AI and heart team recommendations. Traditional models of clinical governance and accountability are not easily adaptable to such scenarios, raising unresolved questions about responsibility and transparency.

Additional concerns relate to patient data privacy and security, as the use of AI often involves uploading sensitive health information to privately operated servers, increasing the risk of unauthorized access or misuse. Furthermore, there is potential for outdated or inaccurate recommendations, given that the computational resources and online updates of these models are not always guaranteed, and offline datasets may become obsolete. These challenges underscore the need for stringent regulatory oversight and robust institutional policies to ensure the safe, effective, and equitable adoption of AI in clinical practice.

From a workflow perspective, careful integration is required to avoid introducing bias into multidisciplinary discussions. Ideally, AI should be interrogated before or after heart team meetings by independent, blinded operators to prevent influencing the collective decision-making process. Clear protocols and prospective studies should also be established for managing discordance between AI and HT recommendations and any potential disagreement that may arise among HT members in consequence of the AI input, in order to prioritize patient safety and maintain transparency. Of note, due to the nature of our study, it was not possible to retrospectively assess inter-observer variability within the HT members for the cases discussed, as such information was not systematically recorded in our institution.

## 5. Conclusions

The integration of AI into clinical decision-making in cardiology holds significant potential, yet its current limitations—both technical and ethical—highlight the need for caution. While AI can assist in standardizing recommendations, as seen in its preference for CABG in multivessel disease, it lacks the nuanced evaluation provided by experienced clinicians. Addressing challenges related to patient data privacy, algorithmic variability, and regulatory oversight will be essential to ensure that AI complements, rather than compromises, clinical judgment in the pursuit of safe and personalized care. Finally, future research should focus on evaluating similar AI models in a range of clinical settings beyond high-volume cardiology centers. Potential areas include emergency medicine (e.g., triage prioritization and resource allocation), primary care (e.g., chronic disease follow-up and risk stratification), oncology (e.g., decision support in multidisciplinary tumor boards), and intensive care units (e.g., early warning systems and clinical deterioration prediction).

## Figures and Tables

**Table 1:** Characteristics of the overall population and subgroups (PCI vs. CABG).

	Overall N = 137	PCI N = 63	CABG N = 74
Age—mean (±SD)	70.3 (±8.6)	73.1 (±9.5)	67.9 (±7.1)
**Clinical presentation**			
CCS—(%)	115 (83.9)	49 (77.8)	66 (89.2)
Stabilized ACS—(%)	22 (16.1)	14 (22.2)	8 (10.8)
Non-invasive stress test—(%)	65 (47.4)	22 (34.9)	43 (58.1)
Concomitant severe VHD—(%)	10 (7.2)	6 (9.6)	4 (5.4)
Obesity—(%)	19 (13.9)	9 (14.3)	10 (13.5)
eGFR—mean (±DS)	78.2 (±22.1)	80.9 (±22.4)	0.578
eGFR < 35—(%)	8 (12.7)	7 (17.1)	0.576
Type 2 DM—(%)	58 (42.3)	29 (46)	29 (39.2)
Hypertension—(%)	124 (90.5)	60 (95.2)	64 (86.5)
Dyslipidemia—(%)	133 (97.1)	61 (96.8)	72 (97.3)
Previous PCI—(%)	22 (16.1)	11 (17.5)	11 (14.9)
Previous CABG—(%)	2 (1.5)	2 (3.2)	0
Carotid stenosis > 50%—(%)	21 (15.3)	5 (7.9)	16 (21.6)
COPD—(%)	14 (10.2)	9 (14.3)	5 (6.8)
Poor social condition—(%)	10 (7.3)	5 (7.9)	5 (6.8)
**Echocardiography**			
LVEF—mean (±SD)	54.3 (±8.2)	51.7 (±6.2)	56.6 (±4.3)
LVEF < 40%—(%)	18 (13.5)	12 (19.2)	6 (8.1)
**Risk Scores**			
SYNTAX—mean (±SD)	25.2 (±7.7)	23.4 (±8.0)	27.1 (±7.1)
EuroSCORE II—mean (±SD)	2.1 (±2.1)	2.6 (±2.8)	1.7 (±1.2)
STS mortality–mean (±SD)	2.1 (±2.2)	2.6 (±2.9)	1.6 (±1.2)
**LM involvement and/or CTO lesions**			
LM/LAD + 1 vessel—(%)	7 (5.1)	1 (1.6)	6 (8.1)
LM disease—(%)	28 (20.4)	8 (12.7)	20 (27)
One CTO lesion—(%)	43 (31.4)	19 (30.2)	24 (32.4)
Two CTO lesions—(%)	12 (8.8)	4 (6.3)	8 (10.8)

This table provides a detailed comparison of the clinical, anatomical, and risk characteristics of the entire study cohort (N = 137) and two subgroups: patients recommended for PCI (N = 63) and those recommended for CABG (N = 74). CCS: chronic coronary syndrome; LVEF: left ventricular ejection fraction; VHD: valvular heart disease; eGFR: estimated glomerular filtration rate (Crockroft–Gault); DM: diabetes mellitus; PCI: percutaneous coronary intervention; CABG: coronary artery bypass graft; COPD: chronic obstructive pulmonary disease; STS Score: Society of Thoracic Surgeons Score; LM: left main; LAD: left anterior descending artery; CTO: chronic total occlusion.

**Table 2:** Performance metrics for ChatGPT’s recommendations on PCI and CABG.

	Versus PCI	Versus CABG
Sensitivity (%)	44.4	82.4
Specificity (%)	82.4	44.4
Accuracy (%)	65.0	65.0
Positive predictive value (%)	68.3	63.5
Negative predictive value (%)	63.5	68.3

This table summarizes the diagnostic performance of ChatGPT in predicting PCI and CABG recommendations compared to the heart team. Metrics include values of sensitivity, specificity, overall accuracy, positive predictive value (PPV), and negative predictive value (NPV) for each treatment modality. PCI: percutaneous coronary intervention; CABG: coronary artery bypass grafting.

**Table 3:** Comparison of characteristics for patients recommended for PCI by HT and ChatGPT.

	HT N = 63	ChatGPT N = 41	*p*-Value
Age—mean (±SD)	73.1 (±9.5)	70.9 (±9.9)	0.261
Male—(%)	51 (81%)	32 (78%)	0.804
**Clinical presentation**			
CCS—(%)	49 (77.8)	31 (75.7)	0.985
Stabilized ACS—(%)	14 (22.2)	10 (24.4)	0.985
Non-invasive stress test—(%)	22 (34.9)	15 (36.6)	0.513
Obesity—(%)	9 (14.3)	4 (9.8)	0.559
eGFR—mean (±SD)	82.8 (±21.8)	80.6 (±21.9)	0.529
eGFR < 35—(%)	12 (16.2)	13 (13.5)	0.625
Type 2 DM—(%)	29 (46%)	12 (29.3)	0.103
Hypertension—(%)	60 (95.2)	34 (82.9)	0.047
Dyslipidemia—(%)	61 (96.8)	39 (95.1)	0.646
Previous PCI—(%)	11 (17.5)	9 (45)	0.616
Previous CABG—(%)	2 (3.2)	2 (4.9)	0.646
Carotid stenosis > 50%—(%)	5 (7.9)	4 (9.7)	0.616
COPD—(%)	9 (14.3)	6 (15)	1.0
Poor social condition—(%)	5 (55.6)	4 (44.4)	0.736
**Echocardiography**			
LVEF—mean (±SD)	51 (±10)	56 (±7)	0.020
LVEF < 40%—(%)	12 (19)	4 (9.8)	0.270
**Risk Scores**			
SYNTAX—mean (±SD)	23.4 (±8.0)	20.9 (±8.3)	0.127
EuroSCORE II—mean (±SD)	2.60 (±2.85)	2.17 (±2.69)	0.449
STS Score mortality—mean (±SD)	2.69 (±2.93)	2.76 (±3.41)	0.917
**LM involvement and/or CTO lesions**			
LM/LAD + 1 vessel involvement—(%)	1 (1.6)	2 (7.3)	0.298
LM disease involvement—(%)	8 (12.7)	3 (7.3)	0.521
One CTO lesion—(%)	19 (30.2)	6 (14.6)	0.115
Two CTO lesions—(%)	4 (6.3)	1 (2.4)	0.659

This table compares the clinical and anatomical characteristics of patients for whom PCI was recommended by the heart team versus ChatGPT. Differences are highlighted using *p*-values for variables such as age, comorbidities, and anatomical findings (e.g., SYNTAX score). CCS: chronic coronary syndrome, LVEF: left ventricular ejection fraction, eGFR: estimated glomerular filtration rate (Crockroft–Gault), DM: diabetes mellitus, PCI: percutaneous coronary intervention, CABG: coronary artery bypass graft, COPD: chronic obstructive pulmonary disease, STS Score: Society of Thoracic Surgeons Score, LM: left main, LAD: left anterior descending artery, CTO: chronic total occlusion.

**Table 4:** Comparison of characteristics for patients recommended CABG by HT and ChatGPT.

	HT N = 74	ChatGPT N = 96	*p*-Value
Age—mean (±SD)	67.9 (±7.1)	70.1 (±8.1).	0.077
Male—(%)	66 (89.2%)	85 (88.5%)	0.894
**Clinical presentation**			
CCS—(%)	62 (83.8)	81 (84.4)	0.975
Stabilized ACS—(%)	43 (58.9)	50 (53.8)	0.418
Non Invasive stress test—(%)	43 (58.9)	50 (53.8)	0.418
Obesity—(%)	10 (13.5)	15 (15.6)	0.700
Type 2 DM—(%)	29 (39.2%)	46 (47.9)	0.256
Hypertension—(%)	64 (86.5)	90 (93.8)	0.108
Dyslipidemia—(%)	10 (13.5)	15 (15.6)	0.700
Previous PCI—(%)	11 (14.9)	83 (13.5)	0.806
Previous CABG—(%)	2 (3.2)	2 (4.9)	
Carotid Stenosis > 50%—(%)	16 (21.6)	17 (17.7)	0.522
COPD—(%)	5 (6.8)	8 (8.3)	0.720
**Echocardiography**			
LVEF—mean (±SD)	56.5 (±8.45)	53.5 (±10.4)	0.043
LVEF < 40%—(%)	6 (8.1)	14 (14.6)	0.194
**Risk Scores**			
SYNTAX—mean (±SD)	26.6 (±7.1)	27 (±6.7)	0.746
EuroSCORE II—mean (±SD)	1.70 (±1.27)	2.09 (±1.95)	0.142
STS Score Mortality—mean (±SD)	1.61 (±1.23)	1.83 (±1.42)	0.291
**LM involvement and/or CTO lesions**			
LM/LAD + 1 vessel involvement—(%)	6 (8.1)	4 (4.2)	0.279
LM disease involvement—(%)	20 (27)	25 (26)	0.885
One CTO lesion—(%)	24 (32.4)	37 (38.5)	0.430
Two CTO lesions—(%)	8 (10.8)	11 (11.5)	0.890

This table compares the clinical and anatomical characteristics of patients for whom CABG was recommended by the heart team versus ChatGPT. Key comparisons include risk scores, comorbidities, and anatomical complexity, with significant differences indicated by *p*-values. CCS: chronic coronary syndrome; LVEF: left ventricular ejection fraction; eGFR: estimated glomerular filtration rate (Crockroft–Gault); DM: diabetes mellitus; PCI: percutaneous coronary intervention; CABG: coronary artery bypass graft; COPD: chronic obstructive pulmonary disease; STS Score: Society of Thoracic Surgeons Score; LM: left main; LAD: left anterior descending artery; CTO: chronic total occlusion.

**Table 5:** Logistic regression analysis for factors likely associated with discordance between AI and heart team (HT) decisions.

	*p*-Value	OR
Age	0.017	1.082
Male	0.094	0.335
Obesity	0.927	1.063
Diabetes mellitus	0.043	2.890
Hypertension	0.932	0.933
Dyslipidemia	0.163	0.159
COPD	0.574	1.593
eGFR ≤ 35	0.043	0.184
Carotid stenosis > 50%	0.515	0.626
LVEF ≤ 40%	0.533	1.710
Non-invasive stress test	0.410	0.636
Previous PCI	0.320	1.858
SYNTAX I	0.138	0.944
EuroSCORE	0.064	1.484
STS Score mortality	0.066	0.587
Two-vessel disease	0.680	0.673
Left main involvement	0.107	0.269

This table presents the results of logistic regression analysis identifying clinical and anatomical factors associated with discrepancies between ChatGPT and heart team recommendations. Key variables include patient age, diabetes status, chronic kidney disease, and anatomical complexity (e.g., SYNTAX score). COPD: chronic obstructive pulmonary disease; CTO: chronic total occlusion; LVEF: left ventricular ejection fraction; eGFR: estimated glomerular filtration rate (Crockroft–Gault).

**Table 6:** Logistic regression analysis after bootstrapping for factors associated with discordance between AI and heart team (HT) decisions.

	OR	*p*-Value	CI Lower	CI Upper
Age	0.072	0.034	0.009	0.192
Male	−0.884	0.200	−3.096	0.629
Obesity	−0.222	0.749	−2.171	1.520
Diabetes mellitus	0.880	0.096	−0.148	2.461
Hypertension	1.153	0.844	−2.076	5.290
Dyslipidemia	−1.074	0.183	−22.966	20.482
COPD	0.339	0.725	−2.220	3.210
eGFR ≤ 35	−1.660	0.019	−21.245	−0.264
Carotid stenosis > 50%	−0.687	0.279	−2.893	0.747
LVEF ≤ 40%	0.929	0.272	−1.014	3.647
Non-invasive stress test	−0.311	0.550	−1.682	0.875
Previous PCI	0.462	0.479	−1.092	2.280
SYNTAX I	−0.042	0.252	−0.150	0.032
EuroSCORE	0.423	0.044	−0.167	1.117
STS Score mortality	−0.564	0.020	−1.463	−0.080
Two-vessel disease	−4.429	0.536	−21.517	2.286
Left main involvement	−0.897	0.292	−4.492	0.746

This table presents the results of logistic regression analysis after bootstrapping, which identifies clinical and anatomical factors associated with discrepancies between ChatGPT and heart team recommendations. Abbreviations as in Table 5.

**Table 7:** Synthetic comparison between the HT and AI in decision-making for patients with multivessel coronary artery disease.

Domain	Heart Team (HT)	Artificial Intelligence (ChatGPT)
**Decision process**	Multidisciplinary discussion integrating clinical, anatomical, imaging, and patient-centered data	Automated text-based analysis using standardized clinical vignettes
**Input data**	Full access to clinical records, direct imaging review, procedural history, and contextual variables	Standardized textual summaries of clinical and anatomical data
**Imaging integration**	Direct review and interpretation of raw imaging (angiography, CT, MRI)	No direct processing of images; relies on operator-provided textual descriptors
**Time per case**	Approximately 25 min (including data collection, case presentation, and team discussion)	Approximately 10 min for vignette preparation, with instant AI response
**Decision scope**	Considers technical feasibility, institutional expertise, patient preferences, and logistics	Limited to variables presented in text; evaluates based on the available literature
**Strengths**	Integrates nuanced, context-dependent factors; adapts to complexity; includes ethical oversight	Speed, reproducibility, scalability, potential to support rapid triage
**Limitations**	Requires coordination and time; may vary with team composition and dynamics	Cannot process raw imaging; may overlook subtle context; dependent on input quality
**Ethical/legal oversight**	Provided by clinical governance and collective professional responsibility	Raises concerns regarding data privacy, accountability, and regulatory standards

The table summarizes key methodological, operational, and practical differences between the two strategies, including the nature of input data, process workflow, integration of imaging, scope of decision-making, strengths, limitations, and ethical or legal considerations.

## Data Availability

Dataset may be available upon reasonable written request.
